# Going Viral: Assessing the Impact of Social Media on Enrollment in a Coronavirus Disease 2019 (COVID-19) Cohort Study

**DOI:** 10.7759/cureus.56096

**Published:** 2024-03-13

**Authors:** Alexander H Hogan, Katherine W Herbst, Carlie Defelice, Noah Schulman, Aaron M Adams, Christopher L Carroll, Juan C Salazar

**Affiliations:** 1 Division of Pediatric Hospital Medicine, Connecticut Children's Medical Center, Hartford, USA; 2 Department of Pediatrics, University of Connecticut School of Medicine, Farmington, USA; 3 Department of Research, Connecticut Children's Medical Center, Hartford, USA; 4 Department of Geography, University of Connecticut, Storrs, USA; 5 Department of Pediatrics, Wolfson Children's Hospital, Jacksonville, USA; 6 Department of Pediatrics, University of Florida College of Medicine, Jacksonville, USA; 7 Division of Infectious Disease, Connecticut Children's Medical Center, Hartford, USA

**Keywords:** covid, pediatric, recruitment, enrollment, covid-19, sars-cov-2, social media

## Abstract

Objective

This study aimed to quantify the effect of social media posts on study enrollment among children with mild coronavirus disease 2019 (COVID-19).

Methods

The primary outcome was weekly study enrollments analyzed using a run chart. A secondary analysis used linear regression to assess study enrollments two days before and after a social media post, adjusted for the statewide pediatric seven-day-average severe acute respiratory syndrome coronavirus 2 (SARS-CoV-2) case rate, social media posting day, and the interaction of these two variables.

Results

In seven months before social media posting, only eight patients were enrolled. One week after social media posting began, the median weekly enrollment increased (0 to 3). In the regression model, neither social media post day nor the pediatric SARS-CoV-2 case rate was significantly associated with enrollment rate. However, the interaction of a post day and the pediatric case rate was significant.

Conclusion

Social media posts significantly increased enrollment among children with mild COVID-19 in a prospective study. This effect was amplified by the presence of high community case rates during the Omicron wave.

## Introduction

In an era of disruption, nothing has been more disruptive than the coronavirus disease 2019 (COVID-19) pandemic. Across the United States, research operations faced limitations, and even as hospitals regained capacity, persistent safety concerns hindered the recovery of research enrollment numbers [1]. Beginning in March of 2021, our research group initiated the enrollment of children into a cohort study investigating biomarkers in multisystem inflammatory syndrome in children (MIS-C). Although we successfully met enrollment targets for hospitalized children, the enrollment of non-hospitalized children with mild acute COVID-19 was notably unsuccessful despite multiple enhancements to our recruitment strategy. To address this issue, we pivoted to advertising via social media.

Previous studies have demonstrated the successful use of social media to improve study enrollment [2-5], particularly in recruiting children, a population traditionally challenging to reach [6-8]. In this study, we report the impact of using non-targeted, organic social media engagement to improve enrollment of children in a clinical research study of children with mild COVID-19 and assess the sociodemographic characteristics of enrollees.

This article was previously presented as a platform presentation at the 2023 Pediatric Academic Society Annual Meeting on April 29, 2023.

## Materials and methods

This retrospective analysis is embedded within an ongoing prospective study aimed at identifying a potential biosignature for MIS-C [9]. Beginning in March of 2021, our research team started enrolling in a prospective study designed to compare biomarkers in children infected with severe acute respiratory syndrome coronavirus 2 (SARS-CoV-2). We included an inpatient cohort consisting of children hospitalized for either COVID-19 or MIS-C along with comparator cohorts representing non-SARS-CoV-2 viral infections and Kawasaki disease (collectively referred to as the "Inpatient Cohort"). Enrollment took place at Connecticut Children's Medical Center in Hartford, CT.

Simultaneously, an outpatient cohort of children with asymptomatic or mild COVID-19 (henceforth "Mild COVID") was recruited via multiple methods described below. Mild COVID participants contacted the study team via a dedicated phone number or email. Subsequently, they underwent eligibility screening, provided consent, and were enrolled in the study. Participants were then sent a REDCap [10] survey to gather demographic information, health status, and COVID-19 symptom data and were scheduled for an in-person salivary collection in Hartford, CT. Our pre-determined sample size of 100 subjects was based on the overarching COVID biomarker study [9]. This study was approved by the Connecticut Children's Medical Center IRB (approval number: 21-004).

Interventions

**Figure 1:**
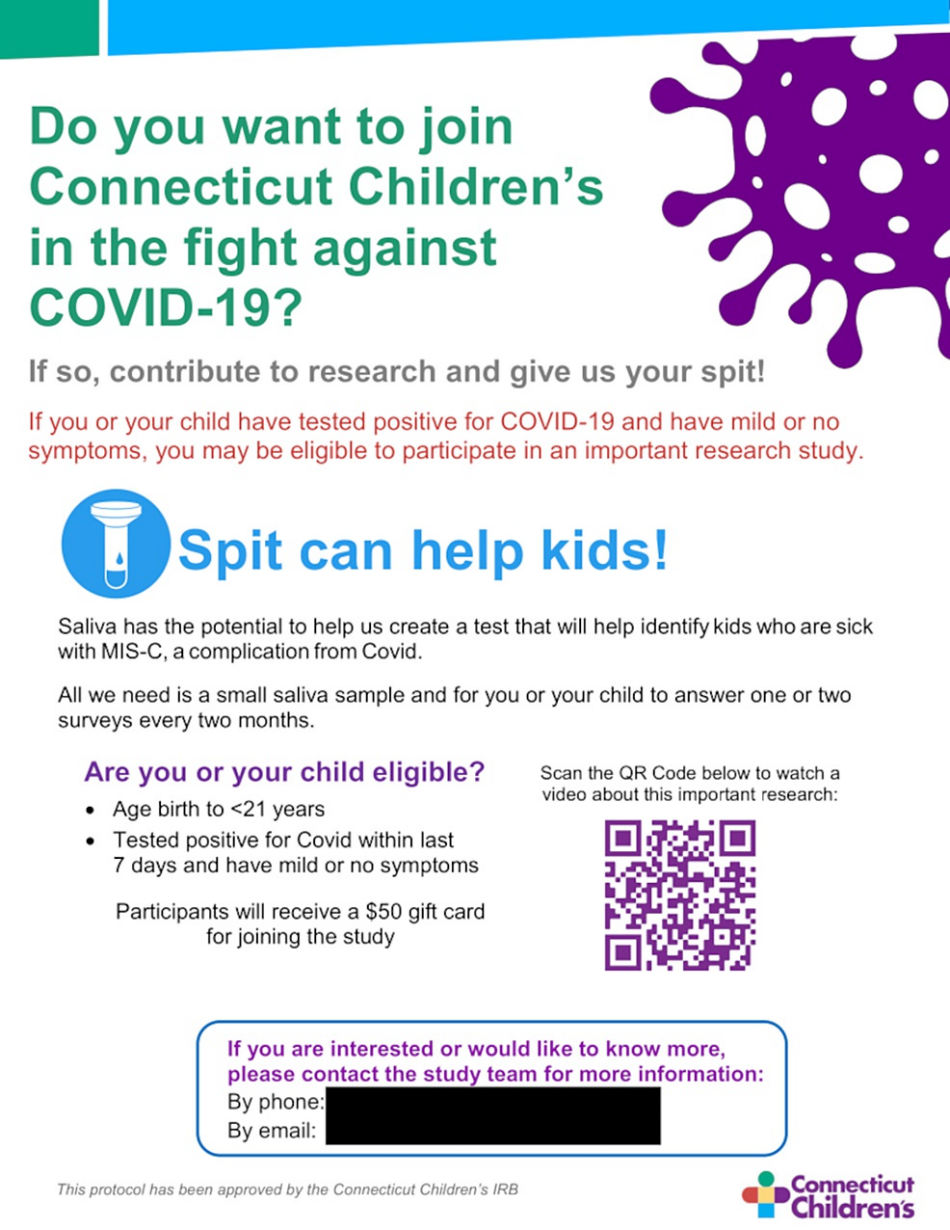
Study recruitment flyer

The initial recruitment of the Mild COVID cohort involved targeted flyer placement in a local university's dormitories specifically designated for COVID-19 quarantined students. Study flyers were also posted in public areas and distributed by student health nurses to students who had tested positive. Critically, study team members could not directly contact students who had tested positive due to university privacy regulations. Therefore, students had to proactively reach out to our research assistants to enroll in the study. Regrettably, for our recruitment plan, the university transitioned to remote learning shortly after we began enrollment. The change to remote learning prompted some students to leave campus, negatively impacting our anticipated enrollment rates. When the accumulation of expected enrollments was substantially below the target in May 2021, we revised our recruitment strategy. These revisions included increasing the enrollment stipend from $5 to $50 and incorporating a quick-response (QR) code into the recruitment flyer that linked to a video providing information about the study and emphasizing the research's significance (Figure 1).

By October 2021, despite the resumption of in-person classes, enrollment remained below expectations, even with a high positive SARS-CoV-2 test rate. In an effort to boost enrollment, we initiated the distribution of flyers at local public schools and two pediatric COVID-19 testing sites. Unfortunately, these interventions did not have a noticeable impact on enrollment numbers.

**Figure 2:**
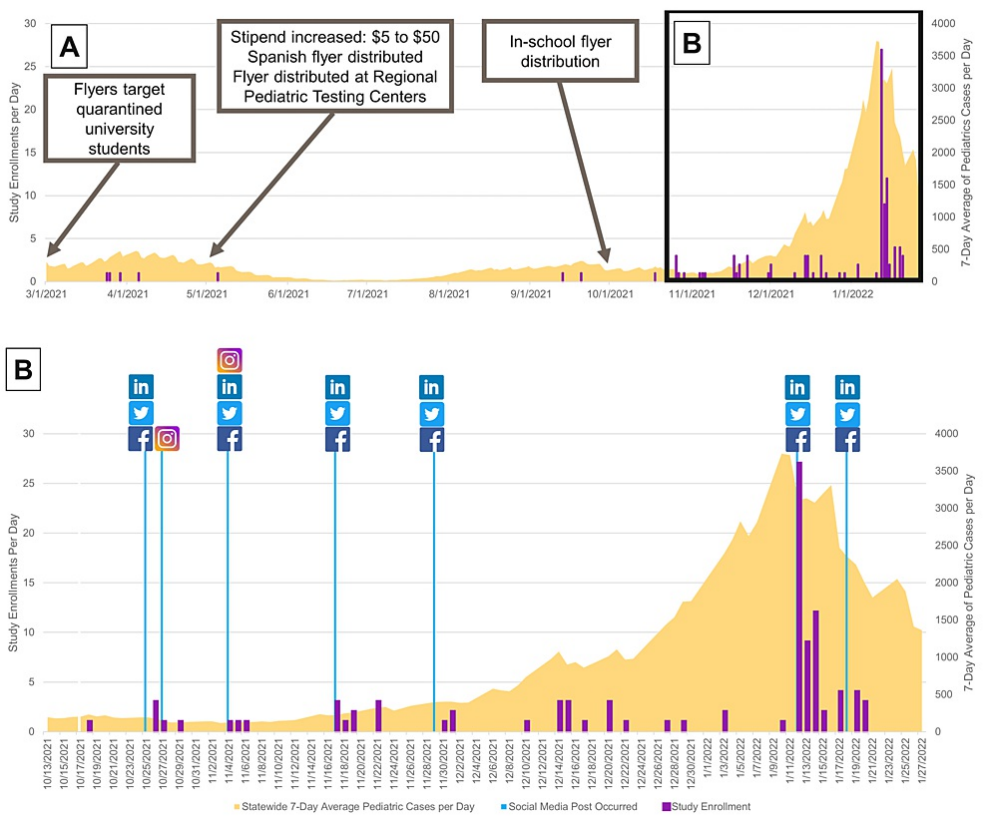
Mild COVID study enrollments from inception to enrollment completion Box A: Purple shows enrollments per day, while yellow shows the seven-day-average pediatric SARS-CoV-2 cases per day in Connecticut in the same time period (March 2021 until February 2022). Box B: Social media post days are displayed with the corresponding social media platform along with the enrollments per day (purple) and average pediatric SARS-CoV-2 cases per day (yellow) from October 2021 to February 2022. SARS-CoV-2: severe acute respiratory syndrome coronavirus 2

Finally, in October 2021, we expanded our recruitment plan to use social media. We obtained IRB approval to share study recruitment information on the hospital's social media platforms. The hospital maintains a social media presence on four platforms: Facebook (24,000 followers), LinkedIn (17,000 followers), Twitter (11,000 followers), and Instagram (9,000 followers). Identical text and images from the IRB-approved recruitment flyers were posted on Facebook, LinkedIn, and Twitter six times over a span of four months (Figure 2). These posts featured images of the approved recruitment flyers (Figure 1).

Study of the interventions

We formulated two analyses to assess the impact of social media posts: (1) a comprehensive evaluation of overall study enrollment and (2) a focused examination of the before-and-after effects following a social media post. In the global analysis, we initially depicted the seven-day-average pediatric community COVID-19 case rate alongside daily study enrollments (Figure 2). This visualization was chosen because study enrollment may be a direct property of community case rates. To quantify the effect of the interventions, we constructed a traditional run chart of weekly enrollments, marked with intervention time points, and analyzed it using traditional run chart rules [11]. 

In the second, more targeted analysis, we compared enrollments two days before a social media post to the number of enrollments two days after a social media post. These counts were assessed by linear regression of Mild COVID enrollments adjusted for the Connecticut seven-day-average pediatric COVID-19 case rate, whether enrollments were before or after a social media post, and the interaction of these two factors. We selected this analysis because we suspected that the relationship between our primary predictor (social media post day) and our outcome of interest (enrollments) likely relied on the community SARS-CoV-2 case rate. We fit the model on a standardized version of the dataset, and 95% confidence intervals (CIs) and p-values were computed using a Wald t-distribution approximation using R version 4.0.3 [12]. P-values <0.05 were considered significant. Sociodemographic variables were analyzed using Wilcoxon's rank-sum, Pearson's chi-squared, and Fisher's exact tests as appropriate.

## Results

**Figure 3:**
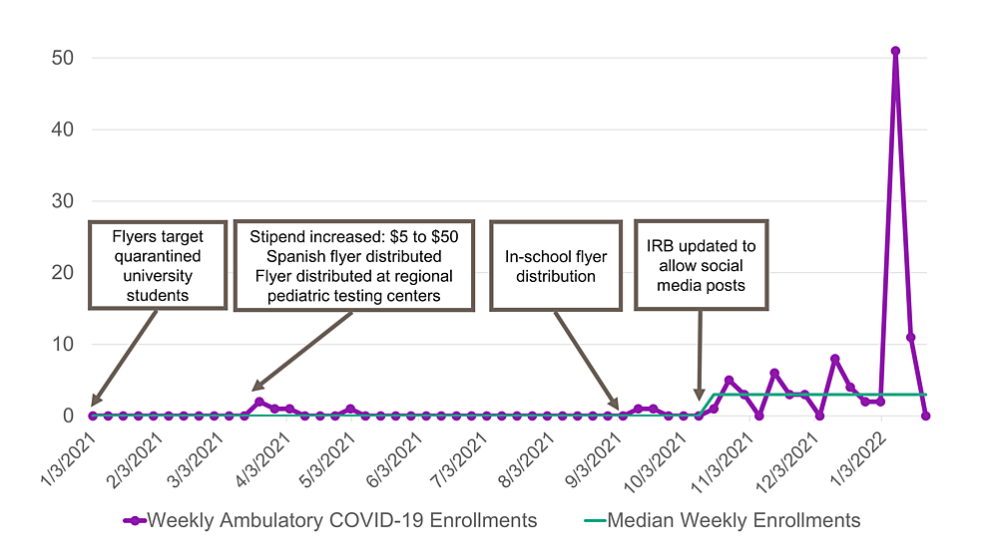
Run chart of weekly study enrollments among children with Mild COVID Run chart of weekly study enrollments among children with Mild COVID. Call-out boxes indicate interventions implemented to increase enrollment. The median line shifted after six points occurred above the prior median the week of October 17, 2021.

The initial recruitment of Mild COVID participants was sluggish. Recruitment was minimally affected by the subsequent interventions to improve enrollments. The first social media post occurred on October 17, 2021. The ensuing posting days and associated platforms are displayed in Figure 2. Within one week of the initial social media posting, there was a signal of nonrandom change [11] in enrollment: median enrollments per week increased from zero to three subjects (Figure 3). A notable demographic trend emerged, with a higher percentage of enrolled subjects in the Mild COVID cohort self-identifying as White (89% vs. 46%; p<0.001), non-Hispanic (88% vs. 61%; p<0.001), and having private insurance (87% vs. 42%; p<0.001) compared to the Inpatient Cohort (Table 1). When respondents who completed the open field (n=86) were asked "Where did you hear about this study?", 81% (n=70) reported "Social Media" (Table 1).

**Table 1:** Demographics of enrolled patients The data has been represented as n (%) and median (IQR) as appropriate. P-values <0.05 were considered significant. IQR: interquartile range

Characteristic	Overall	Inpatient Cohort	Mild COVID	P-value
	N=302	N=192	N=110	
Gender, n (%)				0.64
Female	132 (44)	82 (43)	50 (45)	
Male	170 (56)	110 (57)	60 (55)	
Age in years, median (IQR)	5.8 (1.9-11.2)	5.0 (1.3-10.5)	8.2 (3.5-12.2)	<0.001
Self-identified race, n (%)				<0.001
Asian	14 (4.6)	9 (4.7)	5 (4.5)	
Black	38 (13)	35 (18)	3 (2.7)	
White	187 (62)	89 (46)	98 (89)	
Some other race	36 (12)	35 (18)	1 (0.9)	
More than one race	20 (6.6)	18 (9.4)	2 (1.8)	
Prefer not to answer	7 (2.3)	6 (3.1)	1 (0.9)	
Self-identified ethnicity, n (%)				<0.001
Hispanic or Latino origin	81 (27)	71 (37)	10 (9.1)	
Not of Hispanic or Latino origin	214 (71)	117 (61)	97 (88)	
Prefer not to answer	7 (2.3)	4 (2.1)	3 (2.7)	
Insurance, n (%)				<0.001
Public insurance	116 (38)	103 (54)	13 (12)	
Private insurance	177 (59)	81 (42)	96 (87)	
Prefer not to answer	9 (3.0)	8 (4.2)	1 (0.9)	
"How did you hear about the study?", n (%)				-
Social media	-	-	70 (64)	
No response	-	-	24 (22)	
Physician office flyer	-	-	7 (6.4)	
Sibling enrolled	-	-	4 (3.6)	
Other	-	-	5 (4.5)	

There were substantially more study enrollments on days on, or immediately after, a social media post relative to the previous two days. In total, there were eight enrollments in the two days preceding social media posts and 55 enrollments in the two days following posts. It is important to highlight that this observation was primarily influenced by an outlier enrollment period during the peak of the Omicron wave. During the wave, 36 children were enrolled within two days of a social media post. In our regression model, neither social media post day nor the seven-day pediatric COVID-19 statewide case rate was significantly associated with the enrollment rate. However, the interaction of a post day and the statewide case rate was statistically significant (Table 2).

**Table 2:** Linear regression of study enrollments before and after social media posting, adjusted for statewide pediatric SARS-CoV-2 infection rate *Defined as the number of enrollments on, or the day after, a social media post (days 0, 1) relative to enrollments in the preceding two days (days -2, -1). **Defined as the seven-day rolling average of pediatric cases in Connecticut on, or the day after, a social media post (days 0, 1) relative to enrollments in the preceding two days (days -2, -1). P-values <0.05 were considered significant. The regression output data have been represented as the β estimate, standard error, and p-value. SARS-CoV-2: severe acute respiratory syndrome coronavirus 2

Variable	β estimate	Standard error	P-value
Social media post day*	-1.019	2.969	0.74
Statewide case rate**	0.001	0.001	0.66
Interaction of social media post day and statewide case rate	0.008	0.002	0.001

## Discussion

Enrolling pediatric patients in research has always been challenging [13], and the COVID-19 pandemic exacerbated these difficulties. Our efforts to enroll children with Mild COVID infections were ineffective until we began advertising through social media. There was an increase in median weekly study enrollments immediately after social media posts began, and higher enrollment rates were sustained for 10 weeks. The effect of posts on enrollment was dramatically more pronounced when posts occurred when our area was overwhelmed with pediatric COVID-19 cases during the Omicron wave, as evidenced by the interaction of post day and case rate in the linear model. During the wave, we rapidly met our enrollment goal of 100 children with Mild COVID. However, patients enrolled were not sociodemographically representative of the Inpatient Cohort and by extension the community our hospital serves.

The demographic composition of children enrolled in the Mild COVID cohort was skewed toward non-Hispanic, White participants. This is a common problem in clinical research [14], including prior work using social media to recruit subjects [6,7,15,16]. Our use of organic engagement, compared to paid advertising, is understudied in the published literature. Prior research has successfully used paid advertisements to enroll specific age ranges and people not already following an institution's social media page to improve enrollment [3,6,16-18]. One promising area of research to combat the inherent sociodemographic biases of social media is geospatial targeting to increase minority populations in the sample population [6]. Our digital recruitment flyers were posted on our hospital's social media platforms for free due to budget constraints, so population targeting was unavailable. This may explain the lack of sample diversity present in the Mild COVID cohort.

There are several limitations to this study. We cannot determine the cause of the differences in race/ethnicity and insurance status in cohort enrollments; we can only quantify the disparity. This difference may have multiple causes that may or may not be related to social media. It is challenging to determine the number of enrollments we would have obtained during the Omicron wave without social media. We tried to address this by comparing the two days before a post to the two days after the post, as these time points had similar statewide pediatric case rates, and we adjusted for community case rates in the model. The significant interaction term suggests that the community case rate alone probably would not have accounted for the increased enrollments; instead, it needed to be potentiated by social media posts.

## Conclusions

Our findings indicate a significant increase in the enrollment of pediatric patients with Mild COVID infections in a prospective study focused on COVID-19 biomarkers, particularly evident during the Omicron wave of the pandemic. The expected enrollments during high community infection rates were augmented by social media; however, our reliance on organic engagement through social media platforms resulted in a lack of diversity among enrolled participants, predominantly comprising non-Hispanic, White individuals with private insurance. To enhance the representativeness of future studies, consideration should be given to employing paid advertisements and geospatial targeting, which have shown promise in achieving greater diversity in the pediatric population.

Furthermore, additional research is warranted to comprehend the factors influencing the decisions of parents and children to participate in clinical research, particularly within the context of a public health crisis. Understanding these factors can inform strategies to enhance recruitment and ensure a more comprehensive and inclusive representation in future studies.
